# Supplementary material for: A Critical View of the Application of the APEX Software (Aqueous Photochemistry of Environmentally-Occurring Xenobiotics) to Predict Photoreaction Kinetics in Surface Freshwaters
Source: Molecules. 2019 Dec 18;25(1):9. doi: 10.3390/molecules25010009 (PMC7017383; doi:10.3390/molecules25010009)
Supplement: Supplementary file 1 [file molecules-25-00009-s001.zip › 3.2.4_gcc-4.4.0.zip!/3.2.4_gcc-4.4.0/3.2.4_gcc-4.4.0/doc/octave/html/interpreter/Test-and-Demo-Functions.html]

Test and Demo Functions - Untitled


Next: Tips and Standards,
Previous: Dynamically Linked Functions,
Up: Top


---

## Appendix B Test and Demo Functions

Octave includes a number of functions to allow the integration of testing
and demonstration code in the source code of the functions themselves.

- Test Functions- Demonstration Functions
